# A case study for cloud based high throughput analysis of NGS data using the globus genomics system

**DOI:** 10.1016/j.csbj.2014.11.001

**Published:** 2014-11-07

**Authors:** Krithika Bhuvaneshwar, Dinanath Sulakhe, Robinder Gauba, Alex Rodriguez, Ravi Madduri, Utpal Dave, Lukasz Lacinski, Ian Foster, Yuriy Gusev, Subha Madhavan

**Affiliations:** a Innovation Center for Biomedical Informatics (ICBI), Georgetown University, Washington, DC 20007, USA; b Computation Institute, University of Chicago, Argonne National Laboratory, 60637, USA; c Globus Genomics, USA

**Keywords:** Next generation sequencing, Galaxy, Cloud computing, Translational research

## Abstract

Next generation sequencing (NGS) technologies produce massive amounts of data requiring a powerful computational infrastructure, high quality bioinformatics software, and skilled personnel to operate the tools. We present a case study of a practical solution to this data management and analysis challenge that simplifies terabyte scale data handling and provides advanced tools for NGS data analysis. These capabilities are implemented using the “Globus Genomics” system, which is an enhanced Galaxy workflow system made available as a service that offers users the capability to process and transfer data easily, reliably and quickly to address end-to-endNGS analysis requirements. The Globus Genomics system is built on Amazon 's cloud computing infrastructure. The system takes advantage of elastic scaling of compute resources to run multiple workflows in parallel and it also helps meet the scale-out analysis needs of modern translational genomics research.

## 1 Introduction

### 1.1 Background

The popularity of next generation sequencing (NGS) grew exponentially since 2007 due to faster, more accurate and affordable sequencing [1]. Initial studies were focused on comparing data and analysis results from NGS technologies with those from traditional polymerase chain reaction (PCR) and Sanger sequencing methods. Since then, we have come a long way in understanding how different it is from traditional methods and genome wide association studies (GWAS). The potential of NGS is now being tapped in a wide variety of applications including re-sequencing, functional genomics, translational research, and clinical genomics [2], [3].

Focusing on NGS applications for translational research, the most basic use cases involve comparison of two cohorts — a case and control group with added complexity for longitudinal studies and meta-analyses. Such use cases require medium to large sample sizes, ranging from hundreds to thousands of samples, to be able to derive statistically significant results [4]. As these large-scale genomic studies become a reality, high throughput data storage, management and computation for large sample sizes are becoming increasingly challenging.

Current high performance computing (HPC) solutions in the genomics area involve clusters and grids, which are distributed systems targeted towards users who prefer a command line interface. These HPC solutions are not cheap because they require support and maintenance. University based clusters are shared resources with many competing users. To support maximum usage of these expensive clusters, the jobs are queued, and it becomes a buffer for managing IT capacity. For NGS applications that use medium to large sized samples, researchers would have to wait until enough resources become available; the time needed to complete processing becomes unpredictable. Users could potentially avoid queues by using grids, which are a collection of resources from different locations; but the cost of constructing a grid is high and its architecture and management is complex. Cloud computing leverages virtual technology to provide computational resources to users and this virtualization helps better utilize resources [5]. Its shared computing environment and pay-as-you-go storage can greatly benefit geographically dispersed teams working on the same dataset. There are a number of providers that offer cloud based solutions, some of them include Amazon [6], Google [7], and Microsoft [8]. The need for cloud computing for genomic analysis has been well-described by leaders in bioinformatics and computational biology [4], [9], [10] due to its flexibility, scalability and lower costs. This has been proven by the fact that many medical institutes and centers in the US and around the world have already embraced it [11], [12], [13], [14], [15], [16]. NGS analyses are well-suited for the cloud since data upload (of input files) to an Amazon cloud instance does not incur any extra charge and data download (of output files) becomes relatively inexpensive as only a small percentage of output is needed for downstream analysis [17], [18]. There are several cloud service models: (a)Infrastructure as a service (IaaS) offers compute, storage and network resources as a service, (b)Platform as a service (PaaS) that runs applications on the cloud and hides infrastructure implementation details from the user, and (c)Software as a service (SaaS) that provides software and databases as a service. SaaS eliminates the need to install and maintain the software. It also allows users to run HPCprograms on the cloud through graphical interfaces, and may be a promising solution for NGS analysis for biologists and researchers [5], [19].

While a few large genomics sequencing centers such as the National Institutes of Health (NIH) and major academic centers have developed custom solutions relying on significant investment in local computation infrastructure, an increasing number of universities and academic institutions across the US are facing challenges due to increasing interest and demand from researchers to utilize NGS technology. These small to medium size biomedical research entities neither have the capabilities to implement local computing infrastructures, nor are they able to rapidly expand their capabilities depending on sequencing data management needs. Additionally, there is an increasingly urgent need for adequate software support and management systems capable of providing reliable and scalable support for the ever-increasing influxof NGS data. Some academic centers have been developing customized software solutions, which are often coupled with commercial computing infrastructures such as Mercury [20] utilizing Amazon Web Services cloud via the DNAnexus [21] platform. However there is clearly a lack of standardized and affordable NGS management solutions on the cloud to support the growing needs of translational genomics research.

### 1.2 Existing commercial and non-commercial solutions

Before choosing the Globus Genomics system [22] for our case study, we briefly explored various commercial systems that offer solutions including Partek [23], DNAnexus [21], CLC Bio [24], DNASTAR[25], Maverix Biomics [26], Seven Bridges [27] and Golden Helix [28]. At the time we explored these commercial tools, only a few of these systems had cloud based solutions for large scale batch processing and such solutions were too expensive for an academic center to adopt. Galaxy, however is an open source web based platform for bioinformatics analysis [29], [30]. It provides users with an easy-to-use web interface that allows users to create complex biological workflows by simply dragging-and-dropping tools into its “workflow canvas”. The settings and parameters for each tool can be customized by the user. After upload of data, the workflow gets submitted to their backend analysis server. The completed analysis results can be viewed, published (made public), or shared with other users. Galaxy has an expanding repository of tools in its “Tool Shed” [31]. It provides an extensible framework and allows many software tools to be integrated into the platform. An active community of developers ensures that the latest tools are available through the Galaxy Tool Shed. The biggest advantage of the Galaxy framework is that it automatically and transparently tracks analysis details, and allows results to be documented, downloaded, shared, and published with complete provenance, guaranteeing transparency and reproducibility.

A public Galaxy instance operated by Penn State University [32] allows thousands of users to perform hundreds of thousands of analyses each month. This is a great solution for biologists analyzing small genomes, but the free public resource has data transfer and compute usage limits and hence is not suitable for large datasets. A CloudMan framework helps researchers run their own Galaxy server on a cloud infrastructure [33]. However, CloudMan still requires users to understand the operating complexities of cloud computing, an expertise that most researchers lack. Although Galaxy is easy to use, it has data upload, storage and data manipulation bottlenecks, especially for large datasets. It can analyze only sample at a time, and does not take complete advantage of the elastic cloud compute capabilities (Supplementary File 1a, Supplementary File 1b). This limitation of Galaxy is due to its dependence on a single shared file system. When processing large datasets across distributed compute resources, this limitation represents a significant bottleneck [22].

### 1.3 Motivation

This paper presents a case study for using a cloud based computational environment for the processing and analysis of terabyte scale NGS data. The paper is designed to provide guidance to the users ofNGS analysis software on how to address the scalability and reproducibility issues with the existing NGS pipelines when dealing with very large volumes of translational research data.

Analyzing whole genome, exome, or transcriptome sequencing datafor a large number of human subjects samples requires the ability to transfer data from multiple samples into the analysis system (batchprocessing) and run them simultaneously (parallel processing) so as to complete the analysis in a few hours as opposed to days or weeks on a compute-intensive resource that could scale elastically (i.e.,increasing and decreasing compute capacity in response to changing demand). The Globus Genomics system has these necessary features designed for AWS, and is the focus of this case study.

This case study covers an Amazon cloud based data management software solution for next generation sequencing using the Globus Genomics architecture, which extends the existing Galaxy workflow system to overcome the barrier of scalability. We present three NGS workflows to illustrate the data management and sharing capabilities of the Globus Genomics system, and the novel cloud scheduling architecture that can scale analyses elastically across a dynamic pool of cloud nodes. The NGS workflows involve medium to large scale genomics data presented through the Globus Genomics architecture; providing a fast and scalable solution for pre-processing, analysis, andsharing of large NGS data sets typical for translational genomics projects.

The Globus Genomics system was developed at the Computation Institute, University of Chicago.The Innovation Center for Biomedical informatics (ICBI) at Georgetown University has collaborated with the Globus Genomics team on a pilot project to develop and test several NGS workflows and has summarized our experiences in this paper.

## 2 Methods

### 2.1 The globus genomics system overview

**Fig. 1:**
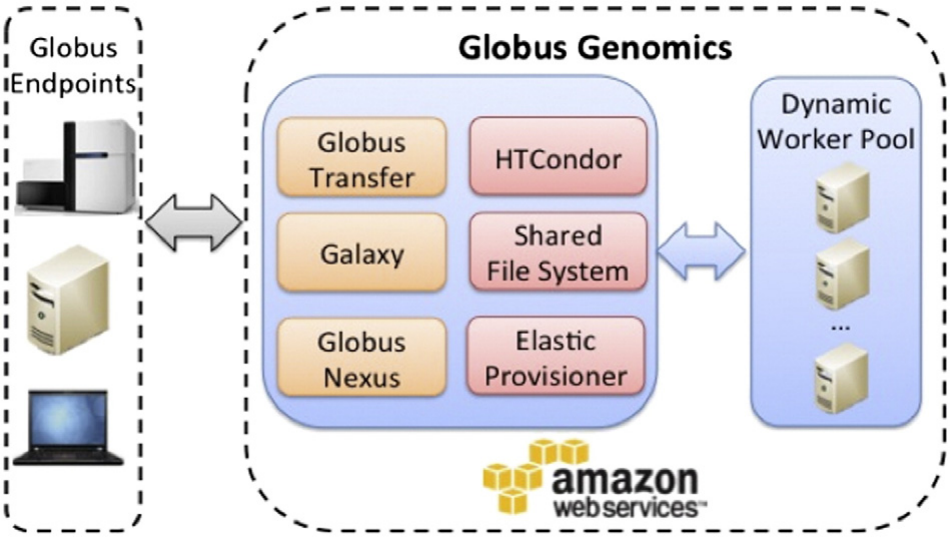
Architecture of the Globus Genomics system. The orange colored components indicate the three distinct components of the system (at a higher level), and the pink colored components are additional features added by the Globus Genomics team.

The Globus Genomics system is a data management and analysis platform built on top of the Galaxy platform to take advantage of Galaxy's best features, and overcome Galaxy's data transfer, storage and data manipulation bottlenecks and limitations. It also provides additional features such as faster computation times, advanced data security, and support and maintenance of the system. It is offered as a Software as a service (SaaS) that eliminates the need to install and maintain the software, and allows users to run HPC workflows on the cloud through graphical interfaces; so users don't have to worry about any operating complexities [22], [34]. By leveraging Galaxy, which is an existing, functional platform with multiple users in the translational research community, the Globus Genomics system maximizes the use of existing capabilities while adding multiple new features that will enable a wider community use, not just for NGS analysis but all other types of datasets as well. Fig. 1 shows a summary of architecture diagram of the system.

#### 2.1.1 How the globus genomics system provides faster computation times

The Globus Genomics system is implemented using Amazon's cloud computing infrastructure. One of the important features of the system is the optimization for selecting the right instance types for the analytical tools. An Amazon web services (AWS) instance type comprises varying combinations of multi-core processors, memory, storage, and networking capacity [35], [36].

As part of the managed service, the Globus Genomics team creates computational profiles for various analytical tools used within the platform to ensure optimal and efficient execution on the AWS. When any new tool is added to the platform, all the critical details required for best performance of the tool, such as the number of compute coresand memory requirements, are collected and documented as a computational profile for that tool. For example, for BWA alignment tool [37], a compute instance with 16 cores and 32 GB RAM was found to provide best performance for the tool. These computational profiles are used to dynamically launch appropriate compute nodes (AWS Spot instances [38]) for a given analytical tool thus making sure the node can run the tool efficiently and within the best possible execution time.

The system takes advantage of elastic scaling of compute clusters using Amazon (Elastic Compute Cloud) EC2 [25]. Elastic scaling refers to the automatic scaling up or down of compute resources based on demand and pre-defined conditions to maximize performance, and minimize costs [39]. The Globus Genomics system provides parallelism at the workflow level, such that multiple workflows can be submitted in parallel, and new compute resources are added to the pool on demand. It also allows tools to use multi-threaded parallelism by launching the appropriate multi-core nodes as per the profile for that tool. The system uses HTCondor [26], a queue based scheduler for efficient scheduling ofthese pipelines over many processors and can run multiple tasks simultaneously for faster computation [34], [40].

#### 2.1.2 How the globus genomics system provides improved data transfer capabilities

Efficient and reliable data transfer is a critical feature in handling large volumes of sequence data. In addition to data transfer, we need robust authentication and authorization mechanisms in place to ensure data security. In order to address these requirements, the Globus Genomics system is integrated with Globus Transfer [41] and Globus Nexus [42] services for transfer and identity and group management capabilities.

Globus Transfer is a service that provides high-performance and secure data transfer between endpoints. An “endpoint” refers to the point where data transfer occurs to and from the Globus Genomics system, and can be a local desktop, data center, external hard drive, or Amazon storage buckets (Amazon S3). Globus Transfer provides managed transfer capabilities (users don't have to wait and manage the transfers and the service provides automated fault recovery), tuning parameters to maximize bandwidth, managing security configurations, and notifications service for error and success [23]. In addition to thetransfers, it also provides sharing capability to share data in place without the overhead of moving data to the cloud. Within the Globus Genomics system, the Globus Transfer service has been integrated with Galaxy using the REpresentational State Transfer Application Programming Interface (REST API). This enables users to perform large-scale data transfers between remote source endpoints and the Amazon cloud where Galaxy is hosted.

The Globus Genomics system leverages the Globus Nexus' identity and group management services. Globus Nexus integration handles the authentication operations ensuring secure access to data. It provides Single Sign On (SSO) across the entire infrastructure and when transferring data to/from other endpoints thus allowing Globus Genomics users to sign in using their preferred identity. Globus Genomics also uses the groups within Globus Nexus to control access to a particular project's instance or to limit access to data, applications and workflows.

User authentication in the Globus Genomics system follows the typical OAuth2 workflow where by a user is redirected to authenticate using Globus Nexus (where they can use their preferred identity provider), and then the user is redirected back to the Globus Genomics instance with a limited time access token which is mapped to the Galaxy session and the Globus username. Thus users don't have to create new account with the Galaxy component and their Globus username is used across various components of the system (Transfer and Galaxy). This mapped information is used by Globus transfer service to perform data transfer on the user's behalf.

Globus Transfer leverages Globus GridFTP [43] an open source, standards-based [44] technology for reliable, high performance, secure data transfer; and its superiority over other technologies has been well-established [45], [46], [47]. Supplementary File 2 shows a performance comparison of a number of data transfer technologies done by the Globus Genomics team.

These Globus platform services are used by many large computing facilities including XSEDE[48], KBase [49], and other national centers including Semel Institute at UCLA,NYU Langone Medical Center, STAR Experiment at Brookhaven National Lab, University of Colorado, and NERSC (National Energy Research Scientific Computing Center) [50]. The 1000 Genomes project [51], [52] and EBI's European Nucleotide Archive [53] now offer data download options using the Globus Transfer system. As of September 2014, there are about 25,000 Globus Platform users that have transferred about 1 billion files which is about 60PBs of data.

#### 2.1.3 Additional features — batch submission

For NGS applications for translational research, it becomes a necessity to be able to process batches of samples together. If the computational infrastructure, storage and data transfer capabilities are not powerful and fast enough, it may take many weeks or months to process NGS data. The Globus Genomics team has implemented a batch submission capability that allows users to submit large batches of samples for analysis in parallel.

Called the “batch submit” workflow, it has been implemented as a Galaxy tool within the Globus Genomics system and leverages Galaxy APIs to submit batches of input sequences. Users are required to complete a tab-delimited file template file for each analytical pipeline, where rows represent the different samples to be submitted and columns represent the parameters to be set at run-time. When “batch submit” is submitted, the desired workflow is executed on each sample in parallel. Using the computational profile, each tool in the workflow is optimized to run in the best available compute node (i.e. compute intensive jobs can be submitted to a multiple core node and memory intensive jobs can be executed on high RAM nodes). Thus, multiple samples can use multiple core nodes in parallel to efficiently execute the analysis. The tool also takes advantage of Galaxy's workflow tracking system, and once the batch is submitted successfully, users can track the analysis of each sample separately in its own history within Galaxy.

Another important feature of batch submission is that the data transfers can also be included as part of the workflows. Thus, there is no need to pre-stage the data and each run in the batch can transfer its own input and output data to and from a remote endpoint using Globus Transfer.

This combination of on-demand cloud computing resources and batch submission capabilities makes the Globus Genomics system a powerful platform for NGS data analysis at scale.

#### 2.1.4 Maintenance and support

The Globus Genomics team has adopted a Software-As-A-Service SaaS [54] delivery model so that researchers can access sophisticated analysis functionality without requiring any software to be installed locally. All interaction with the software occurs through web browsers and APIs. This centrally deployed software is updated, operated and supported, a service provided by the Globus Genomics team.

#### 2.1.5 Taking advantage of the galaxy platform for NGS analysis

The Globus Genomics system not only uses Galaxy's workflow and tracking system, but also its pipeline design tool where new pipelines can be designed by end users and deployed on the infrastructure. The Galaxy tool shed has a comprehensive collection of tools to be able to create a wide variety of workflows.

Upon request by a user, the Globus Genomics team can add tools that are not present in Galaxy's tool shed, so the user can take advantage of the latest tools without waiting for a new release of Galaxy. So wherenecessary, custom pipelines can be developed and deployed forscientists. Even though there is flexibility in creating one's own workflows, there is convenience and time saving in reusing already established public workflows.

ICBI has created and provided three ready-to-use common NGS workflows for a convenient and hassle free experience for the user without having to spend time creating workflows. These computational pipelines are widely used best practices for whole genome, whole exome and whole transcriptome data. Some well-known tools used in the best practices include Tophat [55], Cufflinks [56], RSEM[57], GATK[58], Samtools [59], and others; many of which have been reviewed [60], [61]. These standard workflows include data transfer of raw sequencing files into the system, alignment to genome, variant calling and other steps. The processed output files are variant calls or gene/isoform expression data that can be easily exported from the system and used for biological interpretation and drive hypothesis generation for personalized medicine research.

These workflows have been made public, and can be imported and shared within the Globus Genomics system. To demonstrate usability and efficiency, we ran these workflows on publicly available datasets, evaluated their performance and have made the results public.

### 2.2 NGS analysis using the globus genomics system — a case study

For a typical translational genomics project, DNA or mRNA extractedfrom multiple samples of blood/tissue is subjected to library preparation. The libraries will then undergo, for example, Illumina HiSeq sequencing, which outputs raw data in the form of fastq files. After an investigator obtains the raw sequencing files from the vendor or core lab, a number of processing steps are needed to get meaningful results for biological interpretation.

First, the user would have to manage the large amount of data that would arrive from the sequencing center via hard drives, FTP, or other means, which is a nontrivial task. Secondly, the user would have to determine the processing steps, tools, and the appropriate analysis workflow for a given data type. Even knowledgeable users who are familiar with Unix or Python would have to find a local cluster or a high performance computing environment that could handle such large data, install the tools required, and run the analysis. Depending on the sample sizes and computational power of a local machine, this process would take anywhere from a few days to weeks. And this does not include the time required to identify the appropriate set of tools, install the tools, write the necessary scripts to execute the target workflow and secure the level of resources needed for the eventual analysis. Both a novice or knowledgeable user may not want to bother with these implementation details for translational genomics research; a solution such as the Globus Genomics system can save significant time and cost.

**Fig. 2:**
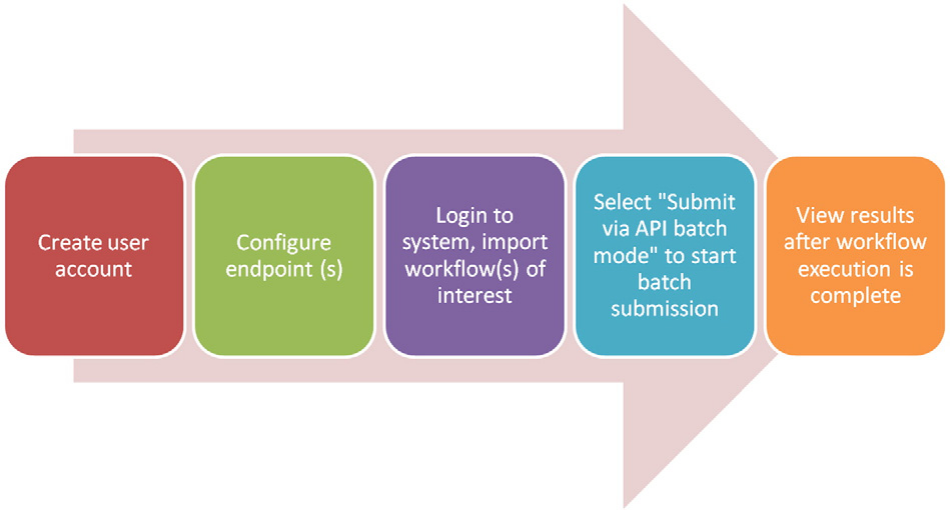
How to run a ready-made NGS workflow in the Globus Genomics system.

In this case study, we ran the three readymade ICBI workflows for Whole exome sequencing (WES) data (b)Whole genome sequencing (WGS) data and (c)mRNA sequencing (RNA-seq) data on the Globus Genomics system on publicly available datasets, and evaluated their performance (cost, time and CPU). Fig. 2 shows what is required of the user to run one of the ready-madeNGS workflows on the Globus Genomics system. Detailed steps are shown in Supplementary File 3a.

The three analytical pipelines are: (a)Whole exome sequencing (WES) workflow (b)Whole genome sequencing (WGS) workflow and (c)mRNA sequencing (RNA-seq) workflow. These workflows are currently designed for Illumina HiSeq platforms. We are currently in the process of creating workflows for other platforms and other NGS data types.

#### 2.2.1 Whole Exome Wequencing (WES) and Whole Genome Sequencing (WGS) workflow

**Fig. 3:**
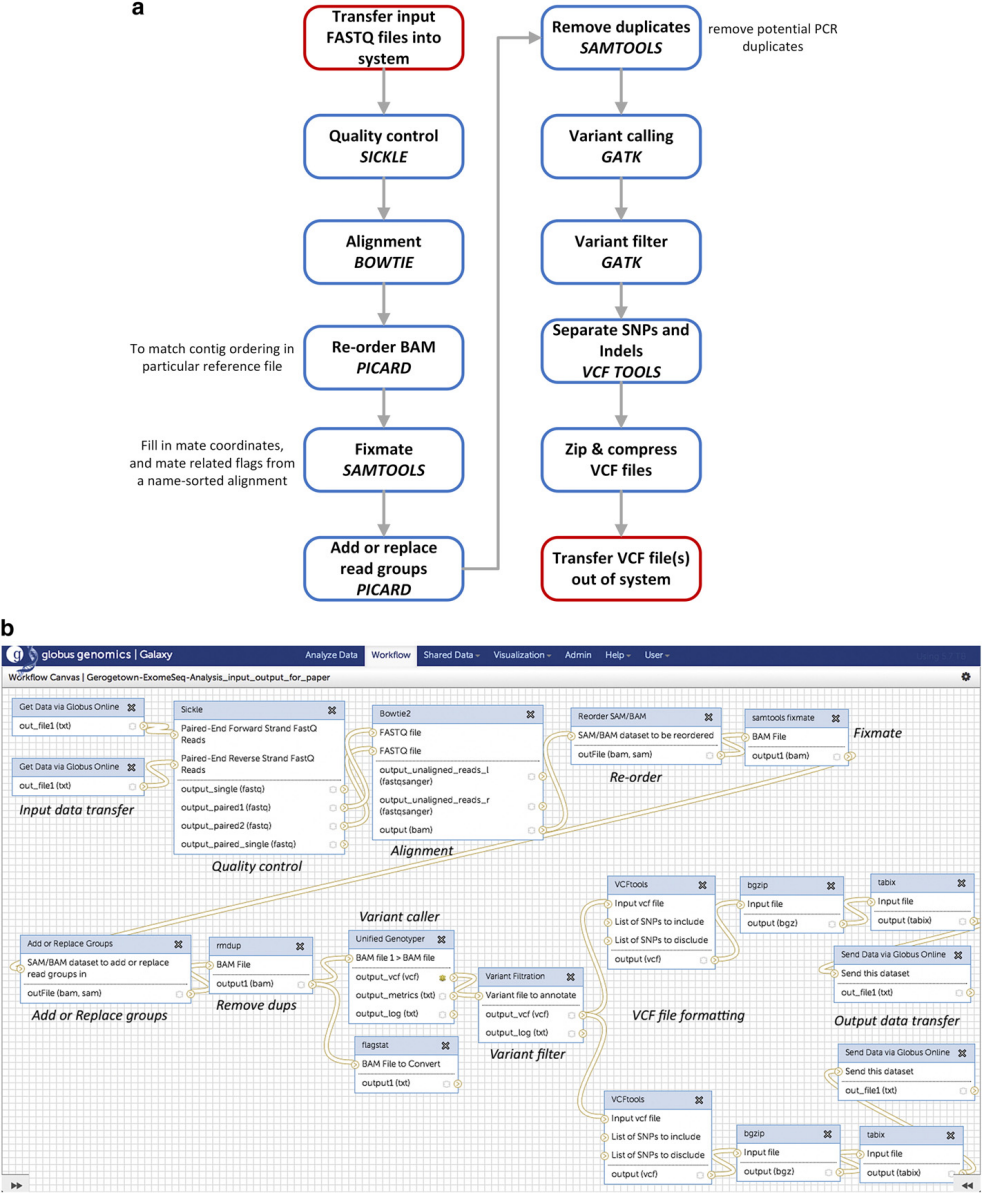
a. Schematic diagram of the whole Genome and whole exome analysis workflow. b. Whole genome and exome analysis workflow inside the Globus Genomics system.

The workflow for pre-processing of WES and WGS is the same, the difference being that WES only sequences the exome region, while in WGS; the entire genome is sequenced as seen in the difference in size and content of the fastq files. (Fig. 3a shows a schematic block diagram of the workflow and Fig. 3b shows the same workflow created in the Globus Genomics system).

The fastq files are filtered based on quality using Sickle [62]. Sickle accepts gzipped fastq files as input and works effectively on paired end data for both WES and WGS data. The filtered output is aligned to a reference human genome using Bowtie2 [63], an ultrafast, memory efficient short read aligner to create alignment files in BAM format. The BAM files are re-ordered and read groups are added using Picard [64]. PCR duplicates removed using Samtools [59]. Variants are called using Genome Analysis Toolkit (GATK) [58]. VCF-tools[65] are used toseparate the SNPs from the indels and produce two variant call format (VCF) files for each sample. These VCF files are small in size (MB range) and can be easily exported from the Globus system. Once exported, the VCF files can be used for further case–control association tests that provide statistically significant variants, which can then be filtered to obtain a short list of non-synonymous, potentially deleterious markers. These variants can then be mapped to genomic regions and further aggregated at the levels of gene, pathways, and biological processes relevant to disease outcome.

#### 2.2.2 Whole transcriptome sequencing (RNA-seq) workflow

**Fig. 4:**
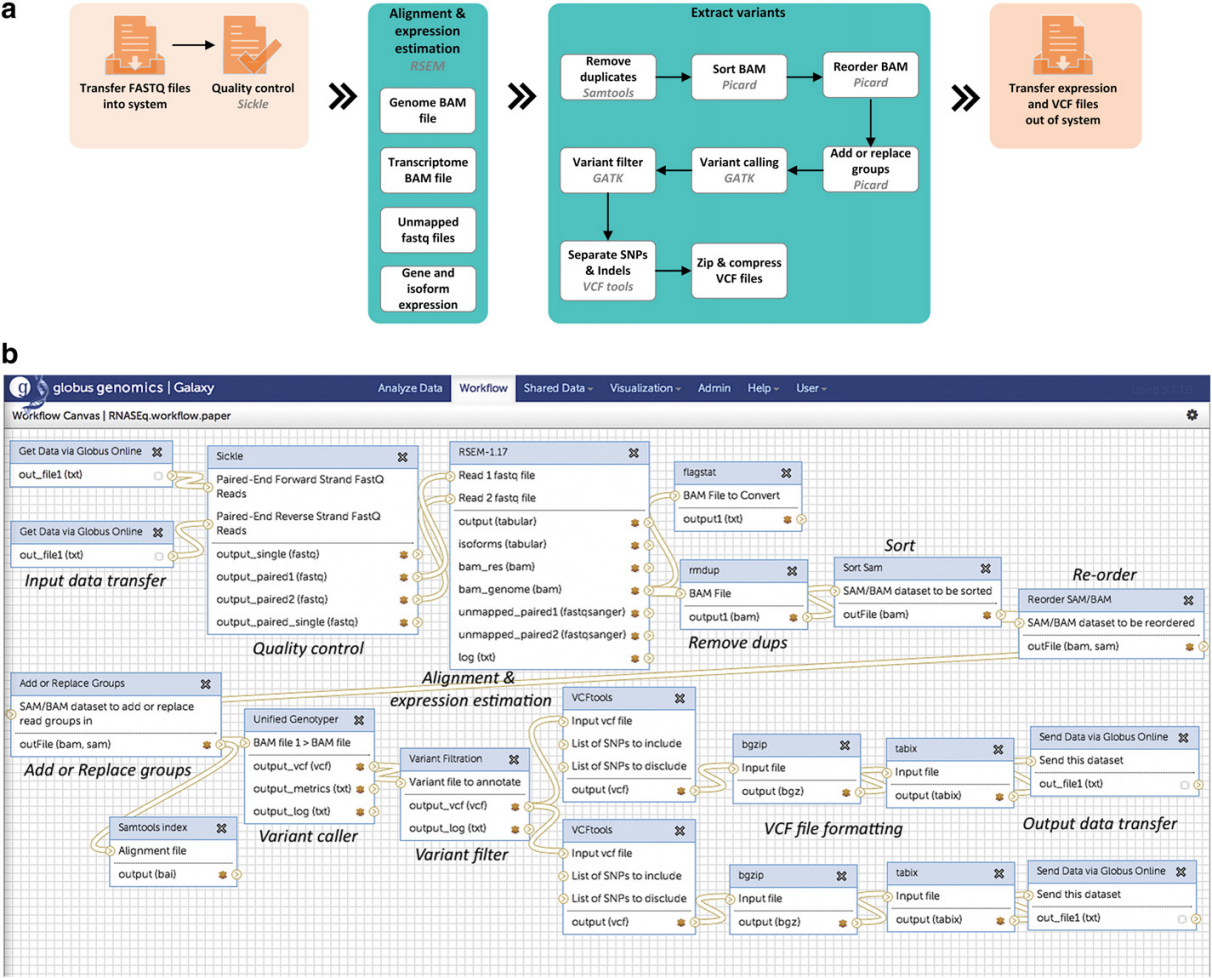
a. Schematic diagram of the whole transcriptome (RNA-seq) analysis workflow. b. Whole transcriptome (RNA-seq) analysis workflow inside the Globus Genomics system.

For this workflow in the Globus Genomics system, RNAseq fastq files are pre-processed for quality checks using Sickle, and input to RSEM[57] a software package that uses Bowtie for alignment and estimates gene and isoform expression levels. Fig. 4a shows a schematic block diagram of the workflow and Fig. 4b shows the workflow in Globus Genomics. Variants are extracted from this data using Picard, GATK and VCF-tools as mentioned above in the form of VCF files. The advantage of variants extracted from RNA-seq data is that these have already undergone transcription and is a validation of variants from WGS data. The output of the workflow are the gene and isoform expression data and the VCF files which can be exported from the Globus system and further analyzed at the level of gene, pathways and biological processes relevant to disease outcome.

For the WES, WGS and RNA-seq workflows created for this case study, the downstream analyses steps have not been included; as the filtering and settings for downstream analysis may vary depending on the biological question in mind. Most of the downstream analysis steps can be added and executed by the user through the Galaxy interface of the Globus Genomics system.

## 3 Results

### 3.1 Performance evaluation

#### 3.1.1 WES workflow performance

We ran the WES pipeline on a batch of 78 samples from a lung cancer study obtained from the European Bioinformatics Institute's Sequencing Read Archive (SRA) [66], from which we downloaded the fastq files.

First, we executed the workflow on a single sample of average input size (6.5 GB compressed paired-end fastq files) to set the baseline, which completed in 4 h. Next, we executed the workflow on all samples, which ran in parallel and completed analysis in 40 h generating between 20–120 GB of data per sample depending on the size of the fastq files. The actual execution time for the batch was about 10 times higher than running a single sample of average input size due to the I/O (disk usage for input/output files) bottlenecks. This bottleneck is introduced by the Galaxy component that requires a shared file system wherein all the jobs from multiple workflows that are run simultaneously need to read the input data from and write the intermediate outputs to the same shared file system [22]. Due to this high I/O nature of the analysis, the Globus Genomics team was able to determine that the servers being used were not optimal for this typeof analysis. They switched to a more I/O intensive node (e.g. h1.4x large) and were able to reduce the total execution time for all 78 samples to about 12 h. The I/O intensive node uses provisioned I/O on the Elastic Block Storage (EBS) [67] when building the shared file system, which significantly improved the read/write performance. Each sample was analyzed in an average time of 10 h, which was closer to baseline. The input data totaled to about 400 GB, and the amount of data generated from running the pipeline was 2.7 TB. The total data handled by the system for this dataset was about 3.1 TB.

**Fig. 5:**
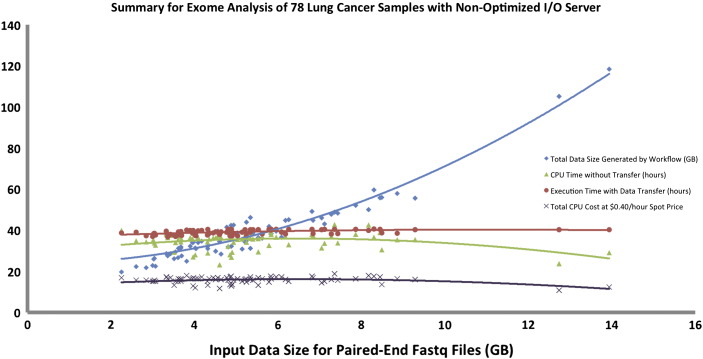
Summary for analysis of 78 lung cancer samples through the exome-seq workflow. Execution time was not optimal due to the high nature of I/O in the workflow. "Spot Price" as mentioned in the figure key refers to the price of the AWS spot instance [38].

**Fig. 6:**
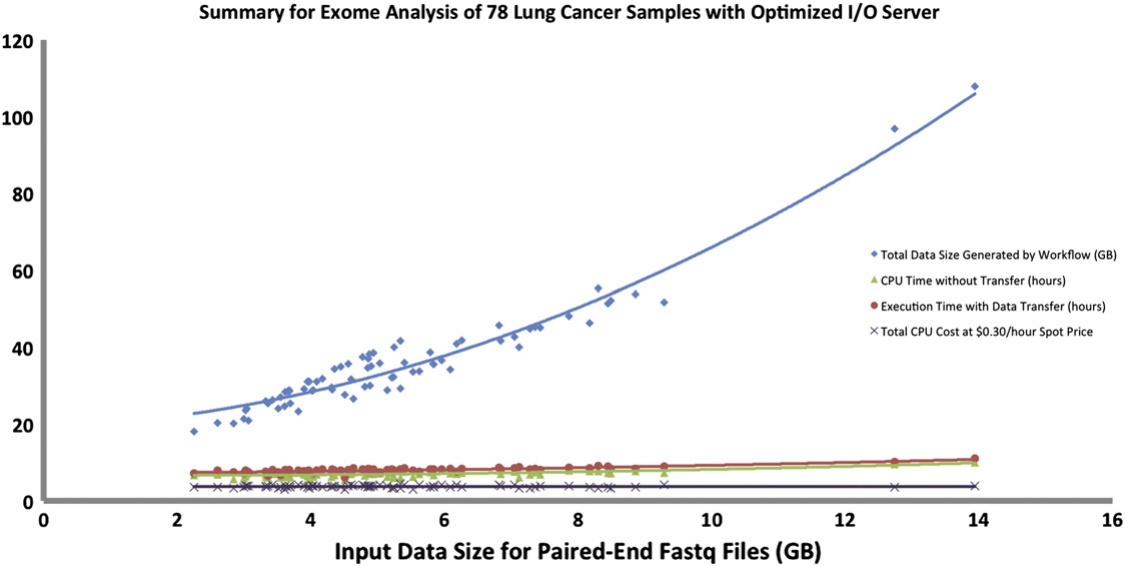
Summary of the 78 lung cancer samples in an I/O optimized server. “Spot price” refers to the price of the AWS spot instance [38].

Fig. 5 shows summary of cost, time and total data generated for the analysis of 78 lung cancer samples through the exome-seq workflow executed on a single multi-core Amazon instance (non-optimal run). Fig. 6 shows summary of cost, time and total data generated for the analysis of 78 lung cancer samples through the exome-seq workflow (optimal run). It shows improvement in CPU and execution time as compared to the non-optimal run. For both figures, we can see that larger input files (fastq files) generate larger intermediate and output sizes, which is typical for NGS analysis.

Supplementary File 4, Supplementary File 5show run times for each sample in the batch job run (non I/O optimized and I/O optimized). It shows a large amount of data generated by intermediate files.

#### 3.1.2 WGS workflow performance

To demonstrate this workflow, we ran the WGS workflow on a human breast cancer cell line dataset. We were unable to obtain fastq files for medium-large sized public WGS dataset on Illumina platform and hence chose this small dataset. This fastq file was of 80 GB size, it took 12 h to produce variants (VCF) files in a compute intensive cluster instance (cr1.8x large). Details of run time for this sample is shown in Supplementary File 6.

#### 3.1.3 RNA-seq workflow performance

We ran this workflow on The Cancer Genome Atlas ' (TCGA's) ovarian cancer samples. We downloaded raw files from the Cancer Genomic Hub (CGhub) archive [68] and extracted fastq files from the raw files. This study has 25 samples in all, and we applied the workflow to 21 samples as 4 samples did not pass quality check. Each sample ran in parallel based on the settings in the computational profiles taking about 20–22 h for each sample to generate expression files and variants, generating about 150 GB of data depending on size of fastq files. The intermediate files contribute the most to the overall size of data. The 21 samples were completed within 24 h from the time the first sample was submitted to the time the last sample completed. Overall, the input data totaled to about 480 GB, and the amount of data generated from running the pipeline is 2.9 TB. The total data the system handled for this dataset was about 3.2 TB.

**Fig. 7:**
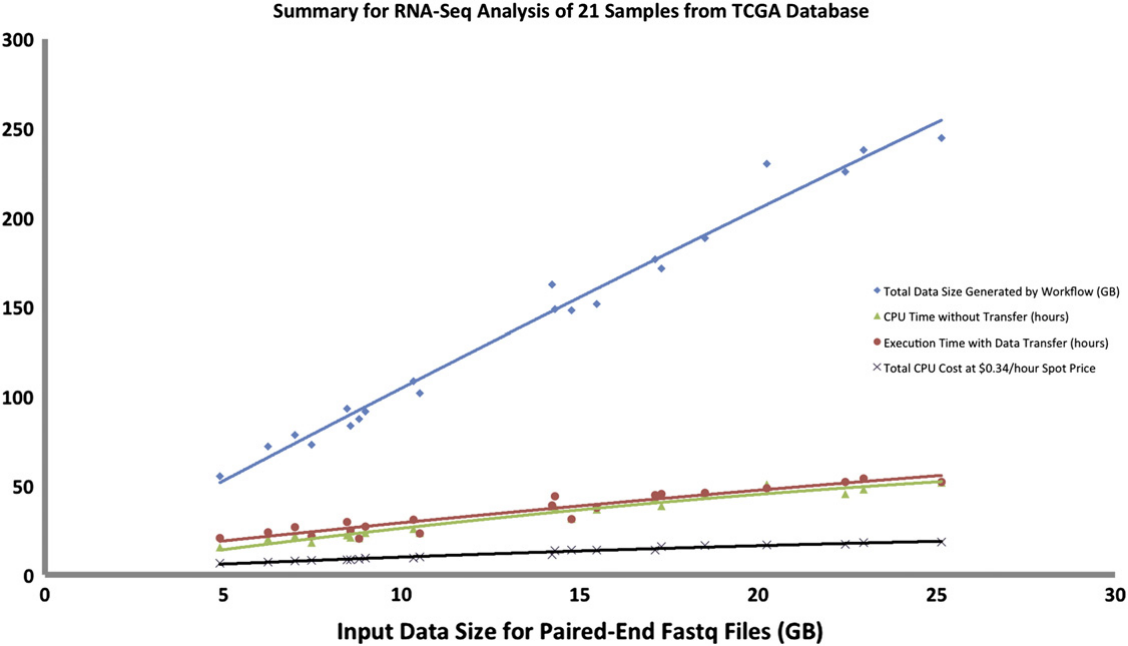
Summary for RNA-Seq Analysis of 21 TCGA samples of varying input sizes. “Spot price” refers to the price of the AWS spot instance [38].

Fig. 7 shows a summary of the RNA-seq analysis for the 21 samples. The Amazon spot instance [38] used for this run (cr1.8x large instance) cost $0.34 per hour. Supplementary file 7 shows run time details for each sample in the batch run.

The graphs in Fig. 5, Fig. 6, Fig. 7show a linear relationship between the input size and data generated by the workflow, while for CPU time, workflow execution time with data transfer, and cost the relationship is non-linear. This is mostly due to heavy I/O utilization especially when multiple samples are written to the same disk space. As smaller samples get completed, the larger samples have less I/O issues and thus can be executed faster. This issue can be resolved by using a more I/O intensive node as previously explained.

## 4 Discussion

In a typical translational research setting a core genomics or a bioinformatics laboratory is facing the challenge of processing and analyzing a massive volume of next generation sequencing data in studies amounting hundreds of DNA or RNA samples. ICBI in collaboration with the Globus Genomics team has conducted a case study aimed at testing a data management solution by running fast, standard, scalable and reproducible bioinformatics pipelines on an enhanced Galaxy platform called the Globus Genomics system built on the Amazon cloud.

### 4.1 User experience from case study

After running the case study at Georgetown ICBI, we found pros and cons with the Globus Genomics system. The main advantage was that the system was user friendly — its user-interface is suitable for scientists who don't have programming experience. The system is especially suited for genomics cores that need to process medium to large volumes of NGS data in a short amount of time, and have to share the processed results with their respective clients. Other advantages of the system include (a)it was convenient to use since it's available on the web, we did not have to worry about updates and maintenance of system, (b)the upload of the file template into the system and batch execution for the analysis of 21 whole transcriptome files and 78 whole exome samples was not difficult, (c)we were able to track the progress in processing of each sample. The workflows could be run overnight without any supervision. Most samples completed processing overnight, which was very convenient as compared to non-cloud based systems.

We found the system to have bottlenecks as well. We had first tested the RNAseq workflow and then the Exome seq workflow. So when wescaled the analysis from 21 samples to 78 samples, we encountered I/O related issues mentioned previously. We learned that the Globus Genomics I/O becomes a bottleneck when multiple concurrent applications start accessing the same file system thus deteriorating the performance. As demonstrated in the results, using provisioned I/O on the EBS[67] when building the shared file system significantly improves the performance. While provisioned I/O can help scale the number of parallel jobs to a couple of hundred, there is a natural upper limit in the number of concurrent jobs that can be handled by the shared file system. The Globus Genomics team is currently working on better load balancing techniques and is working closely with engineers from AWS for larger scalability.

Researchers that have cited the Globus Genomics system include: the Cox lab [69] and Olopade lab [70] at University of Chicago, and the Dobyns lab at Seattle Children's Research Institute [71]. Other users ofthe system include Kansas University Medical Center [72], Inova Translational Medicine Institute, and the Genome Sciences Institute atBoston University [73]. As of September 2014, there are about 20 institutions/research groups actively using the Globus Genomics platform.

### 4.2 Economics of running the analysis pipelines on the cloud

The Globus Genomics team has adopted a Software-As-A-Service SaaS [54] delivery model so that researchers can access sophisticated analysis functionality without requiring any software to be installed locally. Although this model offers cost savings over traditional approaches with multiple local software installations, some costs remain including running the service on Amazon Web Services (AWS), as well as providing any ongoing technical support.

To recover these types of costs, the Globus Genomics team hasadopted a subscription model, whereby users are charged for components of usage such as cloud compute and cloud storage as well as operational and technical support. Fortunately, with the continuous reduction in costs of cloud resources driven by economies of scale and gains in efficiency, public cloud infrastructure becomes increasingly cost effective and most importantly, provides the flexibility of on-demand resource scaling. Advantages for users include lower cost of development as only a single platform is supported, accelerated feature delivery, transparent and frequent software updates, subscription based licensing, pay-as-you-go usage, collaborative and social integration (theoption to publish and rate the workflows, so that other experts or users in the field can also rate these published workflows thus leading to best practices), and intuitive and easy to use interfaces for users.

Table 1 shows actual costs for executing five workflows commonly used in NGS analysis using the Globus Genomics system. To minimize compute costs, the Globus Genomics team created computational profiles of the tools (as described earlier in the System Overview section) used in the analysis workflows and matched them with appropriate Amazon resources to achieve the best price/performance balance during workflow execution. The team also used spot instances [38] to scale-up to the required compute levels with the lowest cost resources.

The Globus Genomics team accounts for AWS storage costs mentioned in Table 1. This allows storage of the computation results for a month, and also accounts for outbound I/O costs from moving the intermediate and final results from Amazon to local resources for downstream analysis or local archiving. While AWS charges for outbound I/O, users can transfer these intermediate and final results of analysis to their own S3 buckets or other AWS storage with no I/O costs, though they may have to pay for the actual storage itself.

At the end, 21 RNA seq samples ran in parallel (average input size 13.5 GB each paired-end set compressed) based on the settings in the computational profiles in about 20–22 h. The total data handled by the system for this dataset was about 3.2 TB. 78 WES samples (average input size 5.5 GB each paired-end set compressed) completed execution on about 12 h. The total data handled by the system for this dataset wasabout 3.1 TB. One WGS cell line sample of 80 GB size completed execution in about 12 h. This will hopefully allow users to roughly predict the time required to complete the analysis given the workflow and size of data.

In summary, the Globus Genomics system achieves a high degree ofend-to-end automation that encompasses every stage of the data analysis lifecycle from initial data retrieval (from remote sequencing center or database by the Globus file transfer system) to on-demandresource acquisition (on Amazon EC2); specification, configuration, and reuse of multi-step processing pipelines (via Galaxy); and efficient scheduling of these pipelines over many processors (via the HTCondor scheduler [74]). The system allows researchers to perform rapid analysis of terabyte scale NGS datasets using just a web browser in a fully automated manner, with no software installation.

### 4.3 Conclusion and future work

The Globus Genomics architecture extends the existing Galaxy workflow system adding not only superior data management capabilities but also a novel cloud scheduling architecture that can scale analyses elastically across a dynamic pool of cloud nodes [22].

We present three NGS workflows for medium to large scale genomic data in a Galaxy based system built on the cloud that executes these workflows across high performance compute systems. We believe that Globus Genomics is a valuable system that provides a hassle free and fast solution for pre-processing and analysis of large NGS data sets typical for translational genomics projects.

We hope to expand this system to support other NGS workflows and platforms in the future. The Globus Genomics team is also developing new features to enable cataloging of dynamic collections of data and metadata including provenance metadata. Another future direction is to provide sophisticated search capabilities to discover and analyze datasets based on user-defined and automatically extracted metadata.

## Funding

This work was supported by in part by the NHLBI grant for Globus Genomics: The Cardiovascular Research Grid [R24HL085343] and by the U.S. Department of Energy under contract [DE-AC02-06CH11357]. We are grateful to the generous support from Amazon, Inc., for Amazon Web Services credits that facilitated early experiments.

## Data access

The results of the analysis shown in this paper can be viewed here: http://icbi-georgetown.globusgenomics.org/ using the following login details — username: testuser@test.com, password: globus. It is a guest account, so users can anonymously access the workflows and analysis results. This is a static instance (not a demo instance) showing the results of the batch jobs run on exome-seq and RNA-seq data. Users can look into the history of each and sample and go through the output of each and every step in the workflow, to demonstrate the transparency, share-ability and reproducibility aspect of the system. Click on Shared Data — Published Workflows to view the workflows demonstrated in this manuscript.Click on Shared Data — Published Histories to view detailed analysis results from the WES and RNASeq batch runs.

The following are the supplementary data related to this article.

Supplementary File 1a Pros and Cons of using Galaxy (user feedback from Twitter).

**Supplementary File 1b:**
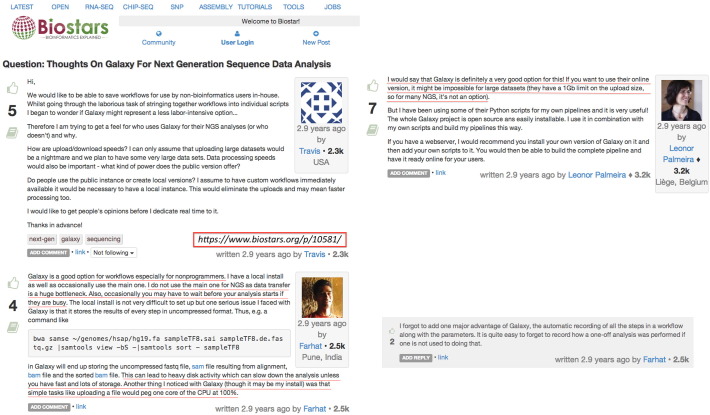
Pros and Cons of using Galaxy from online discussion.

Supplementary File 2 Performance comparison of GridFTP to Aspera and TCP.

Supplementary File 3a How to run an NGS workflow inside the Globus Genomics system.

Supplementary File 3b Example web page with instructions for batch submission.

Supplementary File 4 Per sample execution time for the analysis of 78 lung cancer samples through the exome-seq workflow (non I/O optimized).

Supplementary File 5 Per sample execution time for the analysis of 78 lung cancer samples through the exome-seq workflow (I/O optimized).

Supplementary File 6 Per sample execution time for the analysis of one sample through the WGS workflow.

Supplementary File 7 Per sample execution time for the analysis 21 TCGA samples through the RNA-Seq workflow.

## Figures and Tables

**Table 1:** Sample workflow run costs including compute, temporal storage and outbound I/O^a^.

Workflow	Input data size	Storage size reqs (GBs)	Amazon storage costs	Compute requirement (node hours)	Amazon compute costs	Data download (GBs)	Amazon outbound I/O costs	Total amazon costs
DNA copy number	.070 GB	0.03	<$0.01	0.15	$0.05	0.003	<$0.01	$0.05
microRNA Seq	0.3 GB	1	<$0.01	0.5	$0.17	0.1	$0.01	$0.18
RNA Seq	10 GB (~ 5 Gbp)	70	$0.12	20	$6.80	7	$0.70	$7.62
WES	6 GB (~ 5 Gbp)	50	$0.08	6	$2.04	5	$0.50	$2.62
WGS	72 GB (~ 35 Gbp)	320	$0.53	30	$10.20	32	$3.20	$13.93

a The analysis presented in Table 1 was carried out under the following assumptions: (a)Input data are compressed in GZ format, paired-end Illumina reads (b)RNA-seq analysis includes variant analysis as well: Sickle QC, RSEM (singleton and paired), sort, rmdup, fixmate, picard reorder, picard add or replace groups, GATK Unified Genotyper, GATK recalibration, and GATK variant filtering (c)WES analysis includes: BWA, sort, rmdup, fixmate, picard reorder, picard add or replace groups, GATK Unified Genotyper, GATK recalibration, and GATK variant filtering (d)WGS analysis includes: Bowtie2, sort, rmdup, fixmate, picard reorder, picard add or replace groups, and GATK Unified Genotyper (e)Reference genome used for all analyses is hg19.
