# Supplementary figures and images for: A case study for cloud based high throughput analysis of NGS data using the globus genomics system
Source: Comput Struct Biotechnol J. 2014 Nov 7;13:64–74. doi: 10.1016/j.csbj.2014.11.001 (PMC4720014; doi:10.1016/j.csbj.2014.11.001)

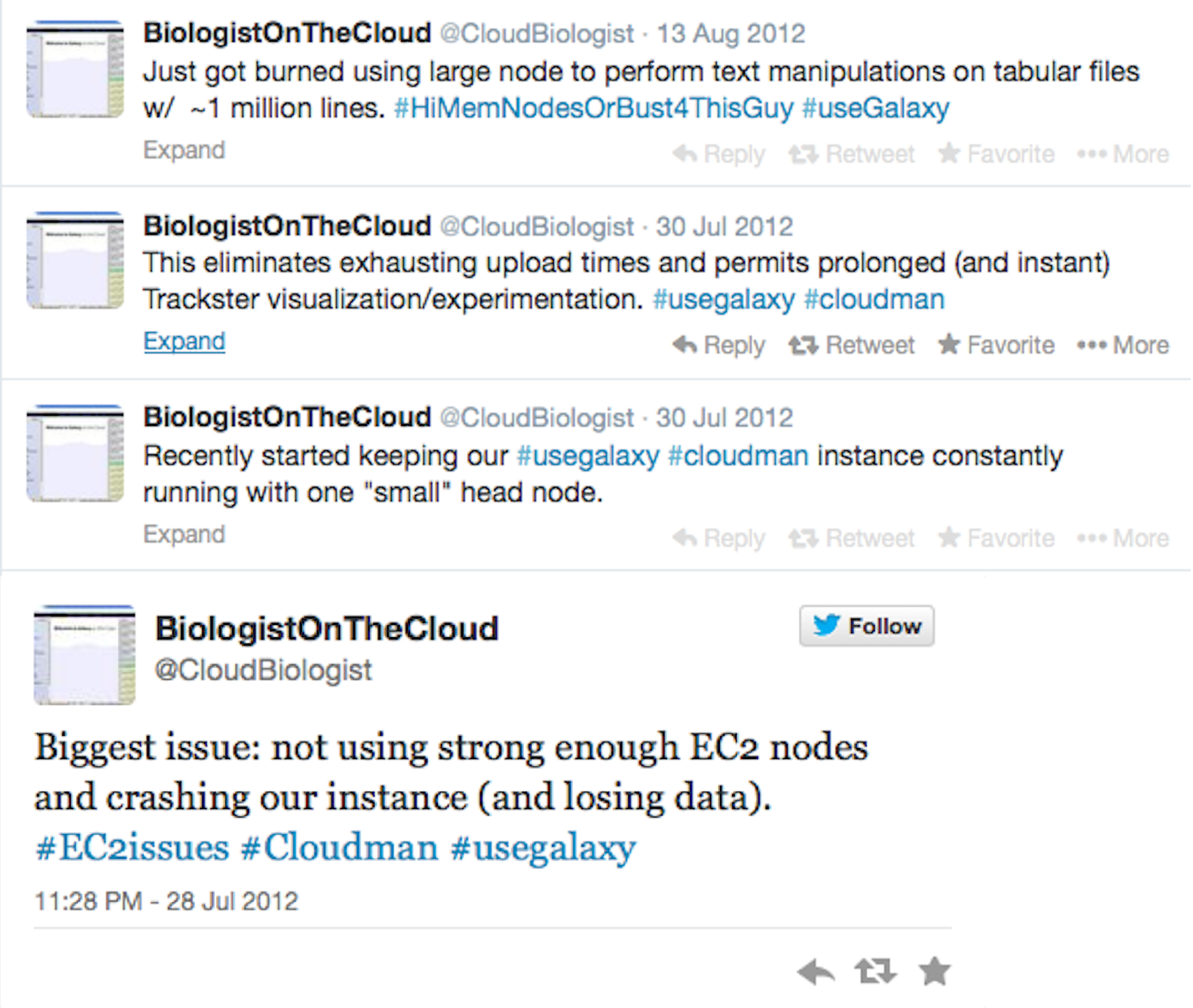

Supplement: Supplementary File 1a — Pros and Cons of using Galaxy (user feedback from Twitter). [file mmc1.zip › CSB00040-mmc1.png]
