# Supplementary material for: A case study for cloud based high throughput analysis of NGS data using the globus genomics system
Source: Comput Struct Biotechnol J. 2014 Nov 7;13:64–74. doi: 10.1016/j.csbj.2014.11.001 (PMC4720014; doi:10.1016/j.csbj.2014.11.001)
Supplement: Supplementary File 2 — Performance comparison of GridFTP to Aspera and TCP. [file mmc2.docx]

# Supplementary File

## Performance comparison of GridFTP to Aspera and TCP*

Globus Transfer leverages Globus GridFTP [[1](#_ENREF_43)] an open source, standards-based [[2](#_ENREF_44)] technology for reliable, high performance, secure data transfer. GridFTP has been heavily used across Grid and High Performance Computing deployments globally for over a decade and is used to move millions of files and over a petabyte of data every day. Data transfer mediated by Globus offers many advantages as compared to commercial products such as Aspera(tm) or bare TCP networks, including higher transfer speeds; freely available endpoint software; standards-based reliability; security; and user friendly management interfaces [3,4].

| **Amazon (US West) 🡪Azure (US West)** | | | |
| --- | --- | --- | --- |
|  | Aspera | Globus Transfer services  (using GridFTP) | TCP |
| **Max (MB/s)** | 62 | 331 | 32 |
| **Sustained (MB/s)** | 48 | 254 | 27.4 |
| **8GB Avg(MB/s)** | 46 | 234 | 24.7 |
| **8GB transfer run #1** | 3m 1s | 34s | 5m 32s |
| **8GB Transfer run #2** | 3m 4s | 39s | 5m 41s |
| **200GB transfer** | 76m | 19m | 2h 21m |

******* *Aspera and GridFTP Servers were provisioned in Azure US West. Aspera was purchased through the Windows Azure Store. Transfers were initiated from both Amazon US West (N. California) and US East (N. Virginia) using a c3.8xlarge instance. Data was transferred to a storage account residing in Azure US West. Multiple test transfers were performed with 8GB and 200GB data sets; the best results from both applications are shown.*

**REFERENCES:**

1. Allcock W, Bresnahan J, Kettimuthu R, Link M (2005) The Globus Striped GridFTP Framework and Server. 54-54.

2. W A (2003) GridFTP: Protocol Extensions to FTP for the Grid. GFD-R-P020, Global Grid Forum; 2003.

3. Mattmann CA, Kelly S, Crichton DJ, Hughes JS, Hardman S, et al. (2006) A classification and evaluation of data movement technologies for the delivery of highly voluminous scientific data products. NASA/IEE Conference on Mass Storage Systems and Technologies (MST 2006): Pasadena, CA : Jet Propulsion Laboratory, National Aeronautics and Space Administration, 2006.

4. Brightwell P (2010) High performance file transfer over IP networks. EBU Technical Review (<http://tech.ebu.ch/techreview):> BBC.
