# Supplementary material for: A case study for cloud based high throughput analysis of NGS data using the globus genomics system
Source: Comput Struct Biotechnol J. 2014 Nov 7;13:64–74. doi: 10.1016/j.csbj.2014.11.001 (PMC4720014; doi:10.1016/j.csbj.2014.11.001)
Supplement: Supplementary File 3a — How to run an NGS workflow inside the Globus Genomics system. [file mmc3.docx]

# Supplementary File

## How a user can run one of the ready-made NGS workflows in the Globus Genomics system

- Create a user account with Globus Genomics at <https://www.globus.org/>
- Configure “endpoint” depending on where the data exists to enable data transfer [https://www.globus.org/globus-connect]
  - If the data exists on a local computer, or external hard drive, the local desktop needs to be configured as an “end point” [ <https://www.globus.org/globus-connect-personal>]. This was the endpoint type used for this case study.
  - If the data exists on a server, the server machine needs to be configured as an “end point” [<https://www.globus.org/globus-connect-server>]
  - If the data exists on the Amazon cloud storage S3, then an endpoint needs to be configured on an Amazon S3 bucket [https://www.globus.org/amazon-web-services/s3-endpoint-configuration ]^1^.
- Log in to the system. Click on Shared Data -> Published Workflows to view the workflows demonstrated in this manuscript. Import the workflow of interest into your account. The workflow is now listed in the “Workflow” tab.
- Data submission
  - If user has only one sample to submit, he/she can directly select the workflow , upload data and execute the workflow
  - Batch submission of input data: - Click on the workflow of interest and select “Submit via API batch mode”.
    - When the user clicks on the link “Submit via API batch mode”, a new web page opens up. This page has information about:
      - API key which the Galaxy system uses to uniquely identify a user. This key can be easily generated by clicking User 🡪 API Keys.
      - How a user can download and complete the template file (contains details about the samples and settings for the tools in the workflow).

An example of such a web page is shown as **Supplementary File 3b**.

- - - Upload the file back to your Globus Genomics instance by clicking the “Analyze” tab 🡪 Get data.
    - Scroll to the bottom of the tool panel on the left, click on the "Batch Submit" tool and select the completed file for the workflow you want to submit. Click on “Execute”
- Click on “User” 🡪 Saved histories to view results after workflow execution is complete. The user will see one new history for each sample. For batch submission, there will be a new history for each row in the text file submitted. The histories are named using a timestamp to make sure they have unique names.

*^1^These instructions may change as this feature is in beta mode. Please refer to* [*https://www.globus.org/*](https://www.globus.org/) *for latest information*
