# Supplementary material for: A case study for cloud based high throughput analysis of NGS data using the globus genomics system
Source: Comput Struct Biotechnol J. 2014 Nov 7;13:64–74. doi: 10.1016/j.csbj.2014.11.001 (PMC4720014; doi:10.1016/j.csbj.2014.11.001)
Supplement: Supplementary File 3b — Example web page with instructions for batch submission. [file mmc4.docx]

# Supplementary File

## Example web page with instructions for batch submission

**Export parameters of workflow for API batch submission**

The Globus Genomics Galaxy API allows submission of a user defined workflow multiple times. You will need to do the following:

1. API Key - You will need to generate an API key to identify yourself with the Galaxy server. This will be a required input for the python script that will submit your jobs. If you don't have an API key, please generate one by clicking on User 🡪 API Keys.
2. Workflow parameters table - You can create a workflow through the workflow generator. You can set which parameters should be set at run time. You can download a skeleton of the table structure you will need to fill out with your input files and parameters that are specific to your workflow. Please don't modify any of the header rows or non-commented lines. You can download the file by clicking on the button below.

Top of Form

 

**Instructions**

The table you have downloaded will help you submit a workflow in batch mode. A workflow can be as simple as running one tool (one process), to having hundreds of tools (hundreds of processes). The structure of the table file downloaded is shown in a figure below. The contents of the file can be separated into three sections:

- - General instructions
  - Workflow metadata - The only parameter the user should modify here is the "project name". All other items in the metadata section should be left alone or the submission process will not work
  - Table data - the header will be supplied in the file. It's important to note that the header row cannot be modified by the user. If so, this will cause errors when submitting the workflow. After the header row, the user should feel free to input the necessary file location, names and parameters for their workflow. The items in the header that will be allowed to be modified are for:
    - Input files required for a tool
    - Parameter values for tools which have been previously set by the user to be filled out at runtime

***The following example should explain what is needed to run workflows in batch mode.***

1. Create an API key if you don't have one yet by following instructions [here](http://wiki.galaxyproject.org/Learn/API#Enabling_the_API).
   - - My API key on my local instance is: 3ab0cce215d049b1a1a2bdfa1eaf6b6b
     - NOTE: This API key will not work on your instance, thus you will need to generate one so that it identifies you and creates the histories under your user account.
2. Create a workflow you wish to submit in batch mode.
   - - [My example is a simple workflow](http://dev.globusgenomics.org/u/arodri7/w/api-batch-test-worfklow) that takes two VCF files, cuts a few columns, adds a column with a user specified text, then concatenates the two VCF files.
     - This workflow in available in the "Shared Data" and "Published Workflows" links in the main menu or [here](http://dev.globusgenomics.org/u/arodri7/w/api-batch-test-worfklow). You should be able to import it to your user account by clicking on the "Import workflow" link.
     - For each tool's input file you will need to create an "Input Dataset" box by searching for the label "Inputs" in the tool panel and selecting "Input Datasets". If this is not specified, you will not be able to specify an input file for that tool.
     - Notice that the workflow has two inputs labeled as Ref1 and Ref2. Adding the "Input Dataset" box tool allowed this to happen.


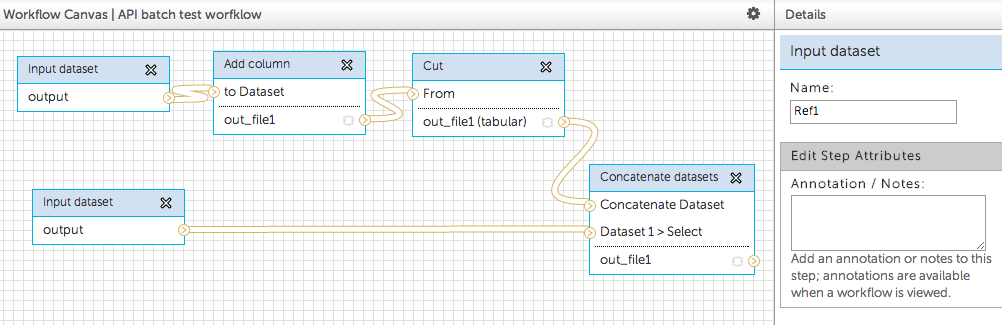


- - - In addition, the "add column" tool has the "Add this value" parameter which has been set to be filled out at runtime. This allows the user to see the column in the batch mode table you will soon download.


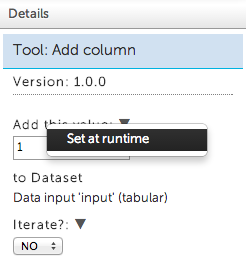


1. On the "Workflows" page click on the workflow you wish to submit in batch mode. A sub-menu will appear. Click on the "Submit via API batch mode" link. The link will take you to a page showing the same instructions you are following.


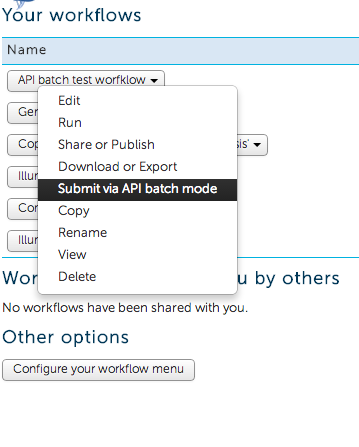


1. Click the ["Export Workflow Parameters for Batch Submission"](http://icbi-georgetown.globusgenomics.org/workflow/parameters?id=50ebc5e997022cba#param_table) button to download the file you will need to edit.
2. Open the tab-delimited file you have downloaded. It should look similar to the figure below.


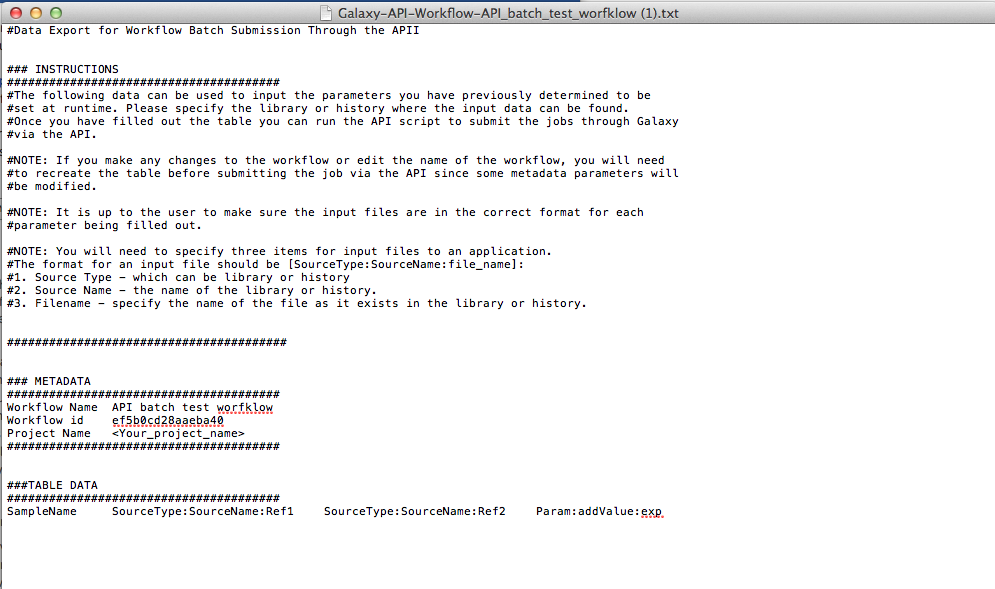


1. Modify the file similar to the figure above by adding the new rows shown. Each row represents a different submission of the workflow. Each submission will be run in parallel. Make sure the columns are separated by a tab character.


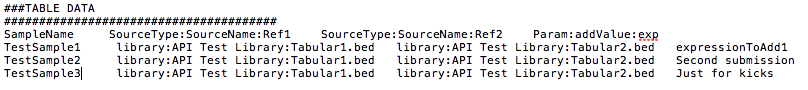


1. Save the file as a text file. Any other type of file will not work.
2. Upload the file back to your Globus Genomics instance.
3. Towards the bottom of the tool panel, click on the "Batch Submit" tool and select the Table file for the workflow you want to submit.
4. Click on "Execute".
5. If you go to the instance and look at your saved histories, you will see one new history for each row you are submitting. The histories are named using a timestamp to make sure they have unique names.


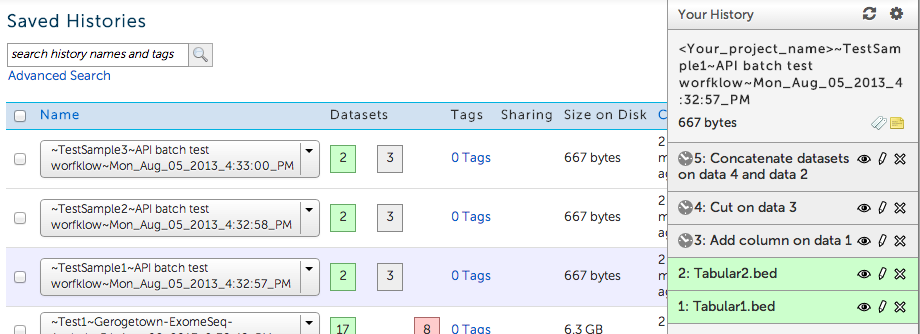


Bottom of Form
